# Exploring the Efficacy and Safety of Levamisole Hydrochloride against *Microcotyle sebastis* in Korean Rockfish (*Sebastes schlegelii*): An In Vitro and In Vivo Approach

**DOI:** 10.3390/ani13111791

**Published:** 2023-05-28

**Authors:** Won-Sik Woo, Gyoungsik Kang, Kyung-Ho Kim, Ha-Jeong Son, Min-Young Sohn, Ji-Hoon Lee, Jung-Soo Seo, Mun-Gyeong Kwon, Chan-Il Park

**Affiliations:** 1 Department of Marine Biology & Aquaculture, Institute of Marine Industry, College of Marine Science, Gyeongsang National University, 2, Tongyeonghaean-ro, Tongyeong 53064, Republic of Korea; 2 Aquatic Disease Control Division, National Fishery Products Quality Management Service, 216, Gijanghaean-ro, Gijang, Busan 46083, Republic of Korea

**Keywords:** levamisole hydrochloride, *Sebastes schlegelii*, *Microcotyle sebastis*, anthelmintic, parasites, monogenean

## Abstract

**Simple Summary:**

Parasitic infections are a major problem in aquaculture, and there is a growing need for alternative treatments. This study investigated the efficacy of levamisole hydrochloride against *Microcotyle sebastis* infections in Korean rockfish. In vitro experiments showed that *Microcotyle sebastis* was sensitive to levamisole hydrochloride, and in vivo experiments tested different administration methods. In a controlled laboratory environment, oral administration has been demonstrated to exhibit efficacy without imposing any adverse effects on fish health. While these findings necessitate further investigation for practical application in actual aquaculture settings, they suggest the potential of levamisole hydrochloride as a therapeutic agent against *Microcotyle sebastis* infections in Korean rockfish. Consequently, this may contribute to the improvement of parasitic control strategies in aquaculture.

**Abstract:**

Parasitic infections pose significant challenges in aquaculture, and the increasing resistance to conventional anthelmintics necessitates the exploration of alternative treatments. Levamisole hydrochloride (HCl) has demonstrated efficacy against monogenean infections in various fish species; however, research focused on *Microcotyle sebastis* infections in Korean rockfish (*Sebastes schlegelii*) remains limited. Therefore, this study aimed to evaluate the efficacy of levamisole HCl against *M. sebastis* infections in Korean rockfish with the goal of optimizing anthelmintic usage in aquaculture. In this study, we first assessed the susceptibility of *M. sebastis* to levamisole HCl in vitro. Subsequently, in vivo evaluations were conducted to assess the drug’s efficacy, safety, and to identify optimal administration methods. In vitro experiments revealed concentration-dependent sensitivity of *M. sebastis* to levamisole HCl, with a minimum effective concentration (MEC) of 100 mg/L. In vivo experiments employed oral administration, intraperitoneal injection, and immersion treatments based on the MEC. Oral administration proved to be a safe method, yielding efficacy rates of 27.3% and 41.6% for 100 mg/kg and 200 mg/kg doses, respectively, in contrast to the immersion and injection methods, which induced symptoms of abnormal swimming, vomiting, and death. Biochemical analyses conducted to assess the safety of levamisole HCl revealed a transient, statistically significant elevation in the levels of glutamic oxaloacetic transaminase (GOT) and glutamic pyruvic transaminase (GPT) on day three post-administration at 20 °C. Following this, no substantial differences were observed. However, at 13 °C, the enzyme levels remained relatively consistent, emphasizing the role of water temperature conditions in influencing the action of levamisole HCl. Our research findings substantiate the efficacy of levamisole HCl against *M. sebastis* in Korean rockfish, underscoring its potential for safe oral administration. These results provide valuable insights for developing parasite control strategies involving levamisole HCl in Korean rockfish populations while minimizing adverse impacts on fish health and the environment. However, this study bears limitations due to its controlled setting and narrow focus. Future research should expand on these findings by testing levamisole HCl in diverse environments, exploring different administration protocols, and examining wider temperature ranges.

## 1. Introduction

Aquaculture, the cultivation of fish, crustaceans, mollusks, and other aquatic organisms for human consumption or other purposes, plays a vital role in global food security. It accounts for a significant proportion of the world’s seafood supply, with approximately 50% of fish and seafood produced worldwide originating from aquaculture [1]. However, the industry faces numerous challenges, including the detrimental impact of parasites on fish health and production [2]. Parasites can inflict various forms of damage, such as stunted growth, elevated mortality rates, compromised immunity, behavioral alterations, and economic losses [3,4]. To mitigate their impact, measures including biosecurity, parasite monitoring and surveillance, integrated pest management, and the application of anthelmintics are implemented.

The Korean rockfish (*Sebastes schlegelii*) is frequently plagued by parasitic infections, particularly those caused by the gill fluke *Microcotyle sebastis*, resulting in substantial damage and considerable economic risk [5,6,7]. For approximately two decades, praziquantel has been utilized as a treatment for *M. sebastis* infections in Korean rockfish [8]. However, there have been reports suggesting the potential for resistance to praziquantel in monogeneans other than *M. sebastis* [9,10]. Consequently, the development of alternative anthelmintics has become imperative due to the documented decline in susceptibility observed in various parasite species [11,12,13,14].

Levamisole, a commercially viable anthelmintic for aquaculture applications, functions as a mimetic of acetylcholinesterase (AchE). It paralyzes or eliminates parasites by depolarizing their ganglia and nerve cells or by interfering with carbohydrate metabolism [15]. Levamisole can be administered via immersion, oral intake, or injection and exhibits immunostimulatory effects when provided orally [16,17,18,19,20]. Research specifically addressing the efficacy of levamisole against *M. sebastis* infections in Korean rockfish is currently non-existent, despite consistent reports of levamisole’s efficacy against monogenean infections in various fish species [18,21,22,23].

The objective of this study was to optimize the use of anthelmintics in aquaculture settings by identifying the most suitable mode of administration and concentration for specific fish species, such as Korean rockfish. Further, this study aims to verify the susceptibility of *M. sebastis* to levamisole in vitro, advance to in vivo experimentation, evaluate the efficacy of various administration methods, including immersion and bath treatments, identify a safe method of administration, and confirm its safety through biochemical analysis. The findings suggest that levamisole HCl demonstrates potency against *M. sebastis* in Korean rockfish and can be safely administered orally. These insights are valuable for the development of parasite control strategies in aquaculture environments.

## 2. Materials and Methods

### 2.1. Investigation of Susceptibility of M. sebastis to Levamisole Hydrochloride In Vitro

In the case of *M. sebastis*, because the culture method has not been established, the Korean rockfish infected with *M. sebastis* was bought from a farm close to Tongyeong-si in Gyeongsangnam-do and transported to the lab. Korean rockfish were used in the experiment, with an average length of 15.5 ± 2 cm and an average body weight of 37.8 ± 3.5 g. Before use in the test, they were acclimatized in a water tank maintained at a water temperature of 20 °C for one week, maintained in dissolved oxygen (DO) of 5.0 mg/L, and then water exchange was performed by 50% every day.

To collect *M. sebastis*, a Korean rockfish that was acclimatized was euthanized using MS222 (Sigma-Aldrich, St. Louis, MO, USA), and its gills were removed. *M. sebastis* was carefully removed while attached to the gill filament using sterilized fine forceps and scissors. The retrieved *M. sebastis* were gently transferred to petri dishes filled with sterile seawater. They were then delicately washed three times with phosphate-buffered saline (PBS) maintained at room temperature to ensure that they remained attached to the filaments. Prior to the in vitro experiments, the parasites were acclimated for 1 h in petri dishes containing sterile seawater maintained at room temperature.

To investigate the sensitivity of *M. sebastis* to levamisole hydrochloride (HCl), ten *M. sebastis* were placed in each well of a 12-well plate containing 1 mL of sterile seawater. Levamisole HCl (Sigma-Aldrich) was dissolved in water as a solvent and added to each well to achieve a final concentration ranging from 1.25 mg/L to 1000 mg/L. The final volume per well in the 12-well plate was 2 mL. An experimental group with solvent added and a control group with no treatment were included. All *M. sebastis* were assessed for their response to levamisole HCl while adhered to the gill filaments, with measurements taken across three replicates for each concentration and the control group. To determine whether *M. sebastis* had detached from the gill filaments or had perished, an inverted microscope, ECLIPSE Ts2 (Nikon, Japan), was employed. Observations were carried out at hourly intervals for a period of 24 h following the commencement of the experiment within the test tubes. The criteria for determining whether the gill filaments had fallen off or died were based on visual observation of the microscope images and reaction to touch with a syringe needle. The concentration at which *M. sebastis* was first killed in the in vitro experiment was determined as the minimum effective concentration (MEC), and then used in the in vivo experiment.

### 2.2. Investigation of Susceptibility of M. sebastis to Levamisole HCl In Vivo

For the in vivo evaluation of levamisole HCl’s efficacy, the Korean rockfish utilized were identical to those used for the collection of *M. sebastis* in the in vitro studies. The concentrations of levamisole used in the in vivo experiments were selected based on the MEC determined from the in vitro studies, with the established experimental groups being 100 mg/kg (the affirmed MEC) and 200 mg/kg (twice the MEC). To compare oral administration, injection, and immersion treatment, each experimental group utilized nine tanks in total, consisting of two concentration groups and one control group for each method. Each tank was filled with 120 L of seawater filtered through sand filters. Ten Korean rockfish were placed in each tank, and the water temperature was maintained at 20 °C. Dissolved oxygen was kept at 5.0 mg/L using an oxygen generator, and the salinity was maintained at 34‰.

In vivo experiments were conducted to assess the effect of levamisole HCl on Korean rockfish infected with *M. sebastis*. The methodology for enumerating parasites in living Korean rockfish was adapted based on previous research on monogeneans [10]. Korean rockfish naturally infected with *M. sebastis* were anesthetized using MS222 (100 mg/L). Following this, forceps and tweezers were utilized to inspect the gill covers and individual gills, enabling the visual counting and recording of adult *M. sebastis* within each aquaculture tank. All Korean rockfish used in the study had a *M. sebastis* count between 10 and 20. The oral administration process was adapted based on prior research aimed at limiting reflux [24]. The prepared levamisole HCl solution was placed into a syringe attached to an oral gavage needle. The anesthetized fish were gently held with hands that had been soaked in seawater and then were vertically aligned for administration of the solution as per the appropriate concentration. Intraperitoneal injections were made through a syringe, dispensing 100 µL of solution each time. For immersion treatment, levamisole HCl was mixed into seawater at the appropriate concentration and the fish were immersed for an hour. The control group, on the other hand, was exposed to fresh seawater for the same duration for immersion treatment, while PBS was used for oral and intraperitoneal injections. All experimental groups were continually monitored for behavioral abnormalities and mortality rates. All experiments were repeated three times.

It is well-documented that the eggs of *M. sebastis* start hatching no earlier than eight days after initial oviposition at a temperature of 20 °C [25]. As a result, we adopted a conservative approach in our efficacy evaluations. Specifically, considering the inherently longer time needed for the drug to reach *M. sebastis* with oral and injection treatments compared to immersion, we evaluated anthelmintic efficacy on the seventh day following administration. During this seven-day period, fish in the oral and injection groups were housed in a recirculating filtration system maintained at 20 °C. They received a daily ration amounting to 0.5% of their body weight and underwent a water change of 50% daily. Conversely, for the immersion treatment group, the anthelmintic efficacy against *M. sebastis* was assessed after a single hour of immersion. Following the completion of the immersion, oral, and injection treatments, we evaluated the efficacy against *M. sebastis* by euthanizing the rockfish with an overdose of MS222, extracting the gills, and separately placing them in petri dishes filled with sterile seawater. Subsequently, the *M. sebastis* specimens were gently removed from the gills using a soft brush, and their numbers were counted using a compound microscope.

### 2.3. Investigation of Blood Biochemical Safety of M. sebastis for Levamisole HCl

The oral administration method was identified as the most suitable mode of administration in the in vivo efficacy evaluation of levamisole HCl. Consequently, a blood biochemical analysis was conducted for safety assessment. The Korean rockfish used in this study were acclimated under the same conditions as those used for in vivo studies. A total of six 120 L tanks were utilized, encompassing both experimental and control groups. Each tank housed 35 rockfish, which were acclimated to the experimental water temperature for a period of seven days. The water temperature in each tank was maintained at either 20 °C or 13 °C, and for each temperature, two experimental groups and one control group were designated. All tanks were filtered using a sand filter, maintaining a salinity of 34‰ and DO levels at 5.0 mg/L. The concentrations of levamisole HCl administered to the experimental groups were the same as those used in previous in vivo studies, namely 100 mg/kg and 200 mg/kg, while the control group received an equivalent volume of PBS. The methods of oral administration for both the experimental and control groups were carried out in the same manner as in the previous in vivo tests.

Sampling was conducted on days 1, 3, 7, 14, and 28 post-administration, during which six Korean rockfish from each of the anthelmintic concentration ranges and the control group were selected for each sampling day. Blood samples were subsequently collected for analysis. A 1 mL syringe was employed to extract blood, which was then transferred to an Eppendorf tube and stored at 4 °C overnight. The serum was obtained by centrifuging the blood sample at 3000 rpm for 20 min. The acquired serum was preserved at −80 °C until further analysis was conducted.

To verify the hematological stability of anthelmintics, a biochemical analysis was conducted on the serum obtained from the sample. The analysis included the measurement of glutamic oxalacetic transaminase (GOT), and glutamic pyruvic transaminase (GPT) using the FUJI DRI-CHEM 4000I device manufactured by Fujifilm, Japan, following the instructions provided by the manufacturer.

### 2.4. Statistical Analysis

All statistical analyses were conducted using GraphPad Prism 9.5.1 software. The anthelmintic efficacy of the tested samples in vitro was determined by comparing the parasite count before and after exposure in the treatment groups, and was calculated using the following formula: Anthelmintic efficacy = (BA − AA)/BA × 100%; where BA represents the mean number of *M. sebastis* in the Korean rockfish experimental group prior to levamisole HCl treatment, and AA denotes the mean number of surviving *M. sebastis* in the treatment group. The evaluation of biochemical analysis between concentrations following oral administration of levamisole HCl was performed based on the Shapiro-Wilk normality test, and the statistical significance was established by conducting a two-way analysis of variance (ANOVA). Statistical significance was denoted according to the following conventions: * *p* < 0.05; ** *p* < 0.01; *** *p* < 0.001. All experiments were repeated three times.

## 3. Results

### 3.1. In Vitro Susceptibility of M. sebastis to Levamisole HCl

**Figure 1:**
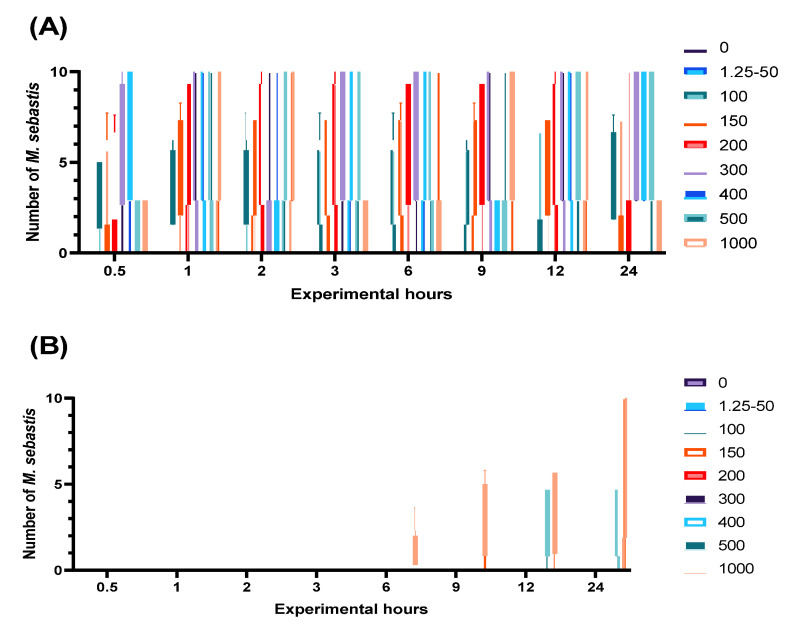
Detachment and death of *Microcotyle sebastis* from the gill filaments upon exposure to levamisole HCl in vitro. (**A**) illustrates the extent of detachment of *M. sebastis* from the gills subsequent to exposure at various concentrations of levamisole HCl. (**B**) depicts the number of *M. sebastis* individuals that were found dead after being exposed to different concentrations of levamisole HCl.

The in vitro susceptibility of the monogenean *M. sebastis*, an infectant of the Korean rockfish, to levamisole HCl was evaluated by exposing the parasites to various concentrations and observing them for 24 h. The count of parasites dislodged from the gill filaments and the number of dead parasites were continually monitored following exposure (Figure 1).

**Figure 2:**
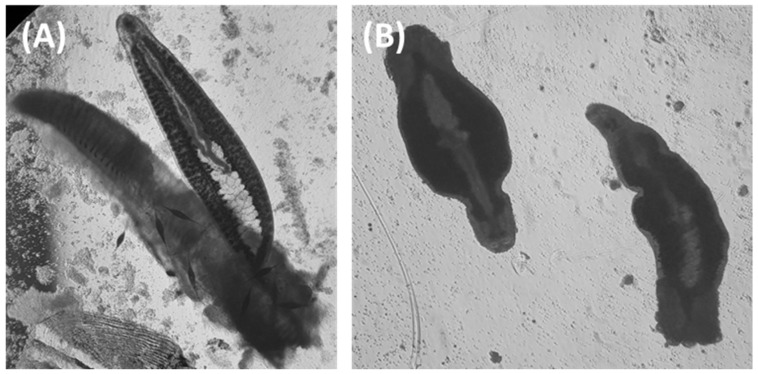
Photomicrograph of *Microcotyle sebastis* exposed to levamisole, observed under an inverted microscope at 40× magnification. (**A**) displays a control *M. sebastis* specimen attached to gill filaments, while (**B**) shows an *M. sebastis* individual that perished as a result of exposure to levamisole.

In treatment groups, no dislodgement or death of parasites was observed during the period of examination up to a concentration of 50 mg/L. However, separation from the gill filaments began to be observed 30 min after drug exposure in *M. sebastis* exposed to concentrations of 100 mg/L or more (Figure 1A). The death of parasites became apparent 6 h after exposure in the group treated with 1000 mg/L, and 12 h after exposure in the group treated with 500 mg/L (Figure 1B). Unlike the control group, dead parasites did not adhere to the gill filaments and displayed contracted body postures upon death (Figure 2). Based on these in vitro findings, MEC of 100 mg/L was determined for subsequent in vivo experiments.

### 3.2. In Vivo Efficacy of Levamisole HCl against M. sebastis

**Figure 3:**
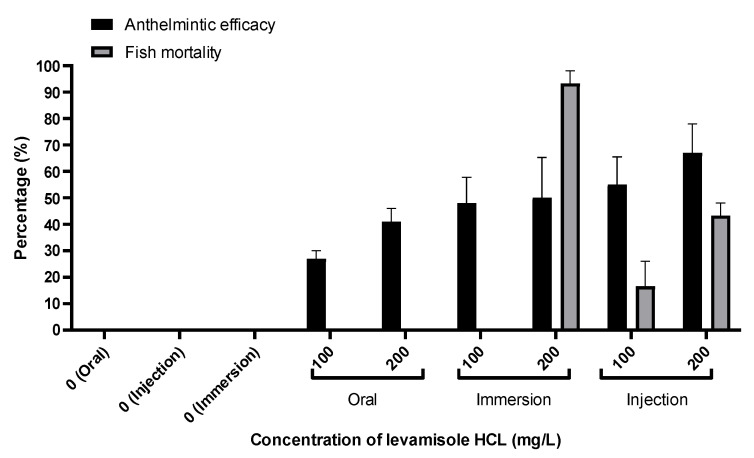
In vivo anthelmintic efficacy of *Microcotyle sebastis* exposed to levamisole HCl via various administration routes.

In vivo experiments were conducted to evaluate the efficacy and optimal administration method of levamisole HCl based on the MEC concentration determined in the in vitro study (Figure 3).

When investigating the injection groups that received 100 mg/kg or 200 mg/kg, efficacy rates of 55.9% and 67.3% were demonstrated, respectively, 7 days after administration. However, abnormal swimming behavior and excessive mucus secretion were observed in all groups. Ultimately, the injection groups exhibited a mortality rate of 16.6% at 100 mg/kg and 43.3% at 200 mg/kg. In the case of immersion treatment, both the 100 mg/L and 200 mg/L groups displayed abnormal swimming behavior, vomiting, and excessive mucus secretion. One hour after immersion, the efficacy rate was 48.9% at 100 mg/L, while the 200 mg/L group, which experienced complete mortality within 20 min, demonstrated an efficacy rate of 50.3%. In contrast, oral administration at doses of 100 mg/kg and 200 mg/kg did not exhibit abnormal swimming behavior and showed efficacy rates of 27.3% and 41.6%, respectively. Based on the in vivo results, oral administration was determined to be the most efficacious method, as it maintained efficacy without adverse effects. Subsequently, the safety of levamisole HCl was confirmed through blood biochemical analysis.

### 3.3. Evaluating the Safety of Levamisole HCl through Biochemical Analysis

**Figure 4:**
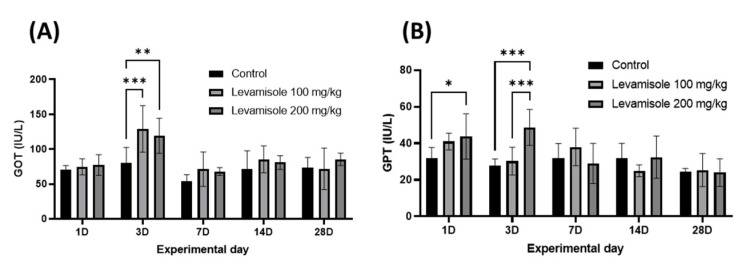
Biochemical analysis of Korean rockfish following oral administration of varying concentrations of levamisole HCl in a 20 °C environment. (**A**) Glutamic oxaloacetic transaminase (GOT) levels, and (**B**) glutamic pyruvic transaminase (GPT) levels. Significant differences are denoted by different superscripts on values (* p < 0.05; ** p < 0.01; *** p < 0.001).

**Figure 5:**
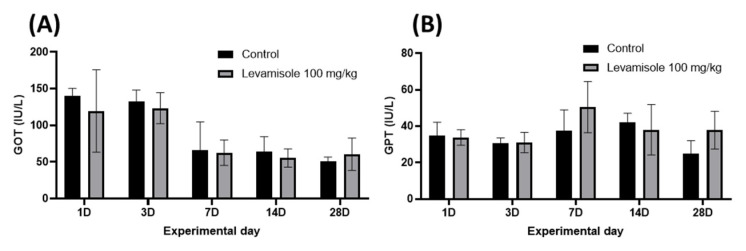
Biochemical analysis of Korean rockfish following oral administration of varying concentrations of levamisole HCl in a 13 °C environment. (**A**) Glutamic oxaloacetic transaminase (GOT) levels, and (**B**) glutamic pyruvic transaminase (GPT) levels.

To evaluate the safety of levamisole HCl in Korean rockfish, serum samples were collected from six fish at 1, 3, 7, 14, and 28 days following oral administration of levamisole HCl at different concentrations and water temperatures. Subsequently, glutamic oxaloacetic transaminase (GOT) and glutamic pyruvic transaminase (GPT) levels were analyzed (Figure 4 and Figure 5). In the GOT analysis conducted after oral administration at 20 °C, both the 100 mg/kg and 200 mg/kg treatment groups exhibited a significant increase on day 3 compared to the control group (Figure 4A). In the GPT analysis, the 200 mg/kg treatment group showed a significantly higher value on day 1 compared to the control group and maintained higher values on day 3, with a significant difference observed between the 100 mg/kg and 200 mg/kg groups (Figure 4B). 

No significant differences were noted on other days. In the GOT analysis performed after oral administration at 13 °C, both the control and treatment groups displayed elevated values on days 1 and 3; however, no significant differences were observed overall (Figure 5A). In the GPT analysis, the 100 mg/kg treatment group showed a higher value than the control group on day 7, but this difference was not statistically significant (Figure 5B).

## 4. Discussion

The aim of this study was to examine the susceptibility of *M. sebastis* to levamisole HCl and to assess the efficacy, safety, and optimal administration method for treating *M. sebastis* infections in Korean rockfish.

In vitro sensitivity testing is a critical approach for the application of therapeutic drugs. It serves to verify the appropriate concentration and potential effects of a drug prior to conducting in vivo research [26]. Establishing the MEC can help prevent excessive chemical usage in aquaculture settings, which could be detrimental to the environment and promote the development of drug-resistant parasites [27]. The evaluation of anthelmintic efficacy is primarily based on their ability to eliminate parasites from the host. This elimination can manifest either as the death of the parasite or a decrease in its activity to a level manageable by the host’s immune system. In the context of extraintestinal parasites, therapeutic success is not exclusively determined by the eradication of the parasite. Anthelmintics like levamisole, which target the parasite’s nervous system, may induce paralysis before death, facilitating the detachment and subsequent removal of the parasite from the infested tissue [28]. Previous research has documented varying sensitivity levels of different parasites to levamisole HCl in vitro. For example, *Neoechinorhynchus buttnerae* demonstrated sensitivity up to a concentration of 108.8 mg/L, while a concentration of 100 mg/L induced mortality in *Gyrodactylus* sp., and 20 mg/L of levamisole HCl exhibited 100% efficacy against *Heterobothrium okamotoi* [29,30]. Moreover, in vitro studies involving parasites infecting *Colossoma macropomum*, such as *Anacanthorus spatulatus*, *Notozothecium janauachensis*, *Mymarothecium boegeri*, and *Linguadactyloides brinkmanni* reported complete mortality within 20 min of exposure to concentrations of 100–125 mg/L of levamisole HCl [23]. 

Our research embarked on an investigation of the effects of levamisole HCl on *M. sebastis*, a parasite prevalent in Korean rockfish. Initial observations indicated that the detachment of *M. sebastis* from the gill filaments upon in vitro exposure to levamisole HCl commenced at a concentration of 100 mg/L. The frequency of such detachments escalated with increasing concentrations of the anthelmintic. Furthermore, *M. sebastis* exposed to levamisole HCl at a concentration of 500 mg/L exhibited symptoms of contraction and paralysis upon death, an outcome consistent with the established modus operandi of levamisole [31]. For a precise evaluation of the impacts of levamisole HCl, in our study, we adopted specific death indicators, namely, the cessation of movement in internal organs and a lack of responsiveness when prodded with a sharp needle [32]. Our approach to designating death diverges from certain preceding studies, which defined death as either the absence of movement or separation from the gills [23,33,34]. We also recorded the absence of movement from the initial stages following detachment from the gills, leading us to postulate that the application of the criteria from earlier studies could result in significantly higher reported mortality rates. In the course of these observations, our study unearthed an intriguing phenomenon: levamisole HCl seemingly inhibited the production of ova across all experimental groups (data not shown). This discovery not only bolsters our primary conclusions, but also delineates new directions for future research into the multifaceted impacts of levamisole. In alignment with previous research, our study underscores the concentration-dependent sensitivity of *M. sebastis* to levamisole HCl in vitro.

Generally, drug administration methods in aquaculture include injection, immersion, and oral administration [35,36]. Immersion requires the direct dissolution of the drug in water, allowing it to contact the entire body mucosa, but necessitates a large quantity of the drug [37]. Intraperitoneal injection primarily targets organs and blood vessels, and although it can be highly effective, it may induce significant stress and prove unsuitable depending on the farm environment [38]. Oral administration imposes the least amount of stress, but the drug’s efficacy may be reduced due to digestive enzymes in the gastrointestinal tract, and its effect may vary based on feed consumption [39]. Therefore, determining the efficacy and safety of each administration method is of the utmost importance [40,41,42].

In this study, we compared three administration methods (injection, immersion, and oral) for their efficacy and safety. The injection and immersion methods demonstrated high efficacy during the experiment; however, they were accompanied by side effects such as abnormal swimming behaviour, excessive mucus secretion, and mortality. In contrast, oral administration exhibited efficacy without side effects, even at high concentrations. Although statistical analysis did not confirm significance, these results underscore the importance of carefully selecting a dosing method that efficaciously treats infections while ensuring the safety of the host organism and demonstrate that levamisole HCl has efficacy against monogeneans such as *M. sebastis*. In previous studies, no mortality or abnormal behavior was observed in *C. macropomum*, even at a maximum oral dose of 1200 mg/kg, and no mortality was observed in *Piaractus mesopotamicus* for oral doses up to 500 mg/kg [18,21]. Based on these findings, oral administration appears to be the most efficacious and safe method for administering levamisole HCl to Korean rockfish. However, further research on higher concentrations of levamisole HCl is warranted. 

Biochemical analysis serves as a crucial indicator for determining the health of farmed fish and assessing safety after drug treatment [43]. Among the various parameters, aspartate aminotransferase (AST) and alanine aminotransferase (ALT), also referred to as glutamic oxaloacetic transaminase (GOT) and glutamic pyruvic transaminase (GPT), are two essential enzymes found in the blood, and are primarily associated with liver function [44,45]. These enzymes remain within a specific range when liver function is deemed normal; however, they leak into the blood and increase in concentration when liver cell damage occurs [46]. In this study, we assessed the safety of orally administered levamisole HCl by investigating changes in the levels of GOT and GPT. Levamisole HCl is rapidly absorbed when administered to fish, with reports indicating that the drug concentration and retention time in the liver, pancreas, and kidneys are relatively longer compared to other tissues [47]. In a prior study, histological changes in the liver were observed in *P. mesopotamicus* after levamisole HCl was mixed with their feed and administered orally for 15 days, with 40–50% presenting dilation at concentrations of 150–300 mg/kg [18]. However, upon assessing the biochemical implications of levamisole in *Cyprinus carpio*, it was discerned that both oral administration and immersion did not instigate any hematological alterations [48,49]. Our findings also revealed significant differences in GOT and GPT concentrations on days 1 and 3 post single-dose administration at a water temperature of 20 °C in Korean rockfish, confirming that levamisole HCl affects the liver. However, subsequent to this, no substantial divergence was observed when compared to the control group. These findings are in line with earlier studies suggesting rapid metabolism of levamisole HCl [31,47].

Water temperature has a direct impact on the metabolism and behavioral patterns of aquatic organisms [50,51]. For effective parasite control, it is crucial to administer treatments according to the specific patterns of both the host fish and the parasite. *M. sebastis* is reported to be most actively infecting at temperatures between 15 °C and 20 °C, which coincides with the typical habitat temperature of Korean rockfish, yet it infects year-round [6,52]. In this study, distinct enzyme level patterns were observed at water temperatures of 20 °C and 13 °C. The enzyme levels at 13 °C were more similar to the control group compared to those at 20 °C. When compared with previous research reporting that serum GPT and GOT levels in Korean rockfish are influenced by water temperature, the findings of this study suggest that a water temperature of 13 °C may be more stable than 20 °C for parasite control strategies involving the administration of levamisole HCl [53]. In light of these findings, it is proposed that a more efficient treatment may be achievable by administering higher concentrations of levamisole HCl at water temperatures lower than the active range of 15 °C to 20 °C for *M. sebastis*, provided that this approach is confirmed to be efficacious.

The cost-effectiveness of therapeutic interventions is a critical factor to consider in aquaculture, yet the potential benefits these treatments offer must not be dismissed [54]. One such treatment is levamisole, an immunostimulant known for its effectiveness across numerous fish species [18,55,56]. However, the specific application context is essential when considering the impact of levamisole beyond its well-documented immunostimulatory properties. Our research contributes to this knowledge base by investigating the efficacy of levamisole against *M. sebastis* in Korean rockfish, thereby expanding our understanding of its role within this host-parasite relationship. The oral administration of treatments, including levamisole, presents an attractive approach for disease management in aquaculture [57,58]. In our study, oral administration demonstrated a comparatively lower efficacy than other methods, but it exhibited greater stability. These findings were obtained under controlled conditions with a single dose at a specific concentration. It is important to note that in a real-world aquaculture setting, anthelmintic would typically be mixed into feed for sustained, oral administration. This approach has not been tested in our study but has shown promise in previous research [18,21,59]. Therefore, further studies are recommended to validate the efficacy of levamisole when administered in this way, considering the specificities of the fish species and the parasite.

## 5. Conclusions

In conclusion, the present investigation elucidates the potency of levamisole HCl in mitigating *M. sebastis* infestation in Korean rockfish, demonstrating a concentration-dependent response and establishing oral administration as the most benign mode among the assessed methods. The results further accentuate the role of water temperature in the bioactivity of levamisole HCl, with a more uniform enzyme profile observed at 13 °C as opposed to 20 °C. These findings contribute towards the formulation of parasitic control strategies, underscoring the potential utility of levamisole HCl as an anthelmintic in Korean rockfish populations, while emphasizing the minimization of adverse environmental and fish health impacts. Nevertheless, the study possesses inherent limitations, primarily its confined laboratory setting, focus on a singular parasite and fish species, evaluation of single-dose administration, and exploration of specific temperature conditions. Future research endeavors should aim to transcend these constraints by investigating levamisole HCl’s efficacy in varied aquaculture environments, against a diverse array of parasites and fish species, utilizing different dosages and administration methods, and across a broader temperature spectrum. Additionally, the observed phenomenon of ovum production inhibition by levamisole HCl in *M. sebastis* merits further scrutiny, potentially paving the way for innovative parasite control strategies.

## Data Availability

The data presented in this study are available upon request from the corresponding author.
